# The Effect of Patient Portals on Quality Outcomes and Its Implications to Meaningful Use: A Systematic Review

**DOI:** 10.2196/jmir.3171

**Published:** 2015-02-10

**Authors:** Clemens Scott Kruse, Katy Bolton, Greg Freriks

**Affiliations:** ^1^College of Health ProfessionsSchool of Health AdministrationTexas State UniversitySan Marcos, TXUnited States

**Keywords:** patient portal, medical record systems computerized, access to information, patient participation, quality, outcomes, meaningful use

## Abstract

**Background:**

The Health Information Technology for Economic and Clinical Health (HITECH) Act imposes pressure on health care organizations to qualify for “Meaningful Use”. It is assumed that portals should increase patient participation in medical decisions, but whether or not the use of portals improves outcomes remains to be seen.

**Objective:**

The purpose of this systemic review is to outline and summarize study results on the effect of patient portals on quality, or chronic-condition outcomes as defined by the Agency for Healthcare Research and Quality, and its implications to Meaningful Use since the beginning of 2011. This review updates and builds on the work by Ammenwerth, Schnell-Inderst, and Hoerbst.

**Methods:**

We performed a systematic literature search in PubMed, CINAHL, and Google Scholar. We identified any data-driven study, quantitative or qualitative, that examined a relationship between patient portals, or patient portal features, and outcomes. We also wanted to relate the findings back to Meaningful Use criteria. Over 4000 articles were screened, and 27 were analyzed and summarized for this systematic review.

**Results:**

We identified 26 studies and 1 review, and we summarized their findings and applicability to our research question. Very few studies associated use of the patient portal, or its features, to improved outcomes; 37% (10/27) of papers reported improvements in medication adherence, disease awareness, self-management of disease, a decrease of office visits, an increase in preventative medicine, and an increase in extended office visits, at the patient’s request for additional information. The results also show an increase in quality in terms of patient satisfaction and customer retention, but there are weak results on medical outcomes.

**Conclusions:**

The results of this review demonstrate that more health care organizations today offer features of a patient portal than in the review published in 2011. Articles reviewed rarely analyzed a full patient portal but instead analyzed features of a portal such as secure messaging, as well as disease management and monitoring. The ability of patients to be able to view their health information electronically meets the intent of Meaningful Use, Stage 2 requirements, but the ability to transmit to a third party was not found in the review.

## Introduction

The 2009 Health Information Technology for Economic and Clinical Health (HITECH) Act placed new requirements on health care organizations in terms of Meaningful Use criteria, which drive reimbursements from the US government for patient-centered care [1]. Appropriate use of patient portals enables health care organizations to meet Stage 2 criteria for patient and family engagement [2]. Despite the advantages of a patient portal, there has not been widespread adoption of this patient-centered tool in the United States [3]. Additionally, research shows that although a provider can make a patient portal available to a patient, it does not necessarily result in a healthier patient [4]. As incentives came to a close at the end of 2014, the authors pondered if there had been any improvement from additional research conducted on the topic.

The US government defines a patient portal as “a secure online website that gives patients convenient 24-hour access to personal health information from anywhere with an Internet connection” [5]. The data are managed by the health care organization, and even the most rudimentary portals enable patients to access information like recent doctor visits, discharge summaries, medications, immunizations, allergies, and lab results. More advanced portals enable patients to request prescription refills, schedule non-urgent appointments, and exchange secure messaging (SM) with their provider [5].

The Meaningful Use criteria are a set of requirements that health care organizations must meet in order to qualify for incentives for the *meaningful* adoption of health information technology (HIT) [6]. Stage 1 criteria focused on data capture and sharing, while Stage 2 (current stage) focuses on advanced clinical processes such as health information exchange and increased patient-controlled data; the latter is specific to patient portals [6].

While most online patient portal programs are still in their infancy, the overall advantage that they provide will need to be benchmarked to determine how to improve not only the flow of information, but to also provide the patient with tools to take part in their care [7]. To be fully utilized in the future, these applications should be implemented to allow for fewer time consuming encounters between patients and providers as well as to enhance the accuracy of information being exchanged.

The ownership of a patient portal distinguishes it from a personal health record (PHR); while the PHR is owned and managed by the patient, a patient portal is owned and managed by the health care organization. A main advantage of the patient portal is that the data are current, while the data in the PHR are current only when the patient updates it. Without a patient portal as an intermediary, the patient would not be able to access the data in the electronic health record (EHR).

Ammenwerth, Schnell-Inderst, and Hoerbst conducted a systematic review on patient portals through a pilot study in 2011 [4]. The authors used medical subject headings (MeSH) terms to focus their research on studies that measured the impact of a patient portal on outcome criteria such as patient satisfaction with the provided care, patient empowerment, costs and resource consumption, mortality, or other relevant clinical parameters. The authors identified 603 papers, 13 of which were experimental or quasi-experimental. Of the 13 papers, five studies were deemed eligible and further analyzed, and four of which were randomized controlled trials (RCTs). Sample sizes ranged from 6-81 participants. A significant flaw in their research was to include the PHR in their search, which, as mentioned above, is significantly different from a patient portal in terms of ownership and management. The features of the patient portal, such as disease management, SM, and the ability to view current personal medical information, are not only key distinguishing details between the patient portal and the PHR, but they also identify features that align with Meaningful Use criteria in Stage 2. Results of this study showed an association between portal use and the following: decrease in office visits rates and telephone contacts, increase in number of messages sent, changes of medication regimen, and better adherence to treatment. The authors summarized their results as a very small effect of patient portals on patient empowerment.

This study intends to duplicate their systemic review with material published from 2011-2014. In light of the HITECH Act, it is expected that patient portals in the current market have evolved to the point that patient empowerment is evident, and medical outcomes can be more readily associated with the use of patient portals. All studies included in the systemic review will evaluate participants, interventions, comparisons, outcomes, and study design (PICOS), as appropriate.

## Methods

**Figure 1:**
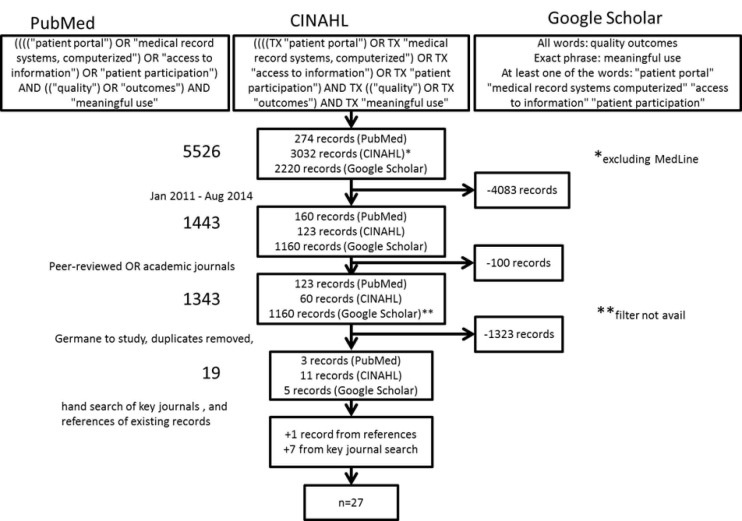
Search criteria and filters by search engine.

The structure and content of this systematic review were loosely adopted from the Preferred Reporting Items for Systematic Reviews and Meta-Analysis (PRISMA) [7]. Three search engines were queried for literature related to patient portals, outcomes (quality), and Meaningful Use. The literature search process, the inclusion and exclusion criteria, and final sample size is illustrated in Figure 1.

MeSH terms from PubMed (MEDLINE) were used as key words in the search. Unfortunately, MeSH does not contain the term “patient portal”. Keywords from the Ammenwerth et al study were all used, with the exception of “health record, personal”. The latter term was not used because of the clear ownership difference between the PHR and the patient portal. As illustrated, Boolean search operators were used to ensure proper terms were used and associated. The three search engines used were PubMed (including MEDLINE), CINAHL (excluding MEDLINE), and Google Scholar.

As depicted in the Ammenwerth et al review, experimental and non-experimental, as well as randomized and non-randomized studies published in academic journals were queried. The RCT and quasi-experimental designs are strong research designs, but we chose a wider array of publications, including those of weaker research designs such as observational studies. In order to be included in our review, publications must have occurred between January 1, 2011, and August 24, 2014. Editorials, government reports, letters to the editor, or non–data-driven studies were not considered, as in the Ammenwerth et al review. Studies for this review must include full text of the article so that the researchers could be certain that the manuscript addressed our research questions. Once studies were identified (N=19), the bibliography/references of each of the chosen articles were reviewed for seminal research otherwise missed. This search yielded one additional article. A key-journal search was also performed in the *Journal of Medical Internet Research*, because it is a prolific publisher of innovative research. This search added six studies and one review, for a final sample size of 27.

Rejection criteria comprised the following. Studies used in this review must have evaluated patient portals used by patients, access to information by patients, or patient participation (in medical decision making). Papers about PHRs or those that confused the line between portals and PHRs were rejected for aforementioned reasons. Studies presented at conferences but not published in peer-reviewed or other academic journals were rejected. The Ammenwerth et al review was not included because we were trying to update their review, and we did not want the results of their review to skew the results of our own.

There were no human subjects in this study; all information came from secondary data sources. The studies used in this research were sources that were publically available, and the subjects could not be identified either directly or through identifiers linked to the subject. This qualifies under “exempt” status in 45 Code of Federal Regulations 46. Therefore, Institutional Review Board review was not required, and consent from subjects was not applicable.

## Results

### Overview

As depicted in Figure 1, 5526 results from the initial search were narrowed down to 19 data-driven studies. From the references of the 19 studies, one additional study was identified. From the targeted-journal search, six studies and one review were added [8-34]. A brief summary of each of the 27 final manuscripts was compiled for analysis and is presented in Multimedia Appendix 1. Results from the searches are generally organized by year of publication. Approximately 22% were published in both 2011 and 2012, 37% were published in 2013, and 41% were published in 2014. Multimedia Appendix 2 provides an in-depth analysis of the studies, interventions, controls, outcomes, populations, and years conducted.

The studies from 2011 that were reviewed covered a wide range of objectives, and all were non-experimental. Goel et al analyzed age and race among portal users [8]. Nijland et al analyzed barriers to use of the patient portal [9]. Horvath et al used a much larger sample to evaluate the association between portal users and adherence to clinic appointments [10]. Results from these studies identified the demographics most commonly associated with use of the patient portal, that the primary barrier to adoption is lack of Internet use, and that the odds of arrival at an appointment increased 39.0% for portal users relative to nonusers of the portal.

In 2012, two of three studies were non-experimental. Palen et al conducted a retrospective study on portal enrollees to associate their rate of use of medical facilities [11]. Urowitz et al identified themes for appropriate use of the patient portal [12]. Debalco et al measured the frequency of access of provider notes by patients [13]. The latter study was able to record significantly positive, clinically relevant benefits by using a patient portal, but the study stopped short of measuring the positive benefit.

Ten studies were reviewed from 2013. Osborn et al used mixed methods to identify demographic differences between portal and non-portal users [14]. Portal users also noted greater medication adherence, particularly for those individuals with chronic illnesses like diabetes. Providers did not perceive a significant increase in workload. Wade-Vuturo et al reported greater patient engagement through the use of the portal [16]. Patients felt that medical decision making was more collaborative between them and their providers, increasing their sense of autonomy.

Several studies from 2013 evaluated the use of the secure messaging feature of a patient portal [15,16,20,22,23]. These studies all demonstrated a high level of patient satisfaction with the feature, and the users did not feel the process to exchange SMs was too complicated. Common to these studies was the perception of high-quality care, better patient-to-provider communication, greater levels of patient education, and a high level of patient engagement/empowerment.

Studies from 2013 also demonstrated several barriers to use of the patient portal; most common were lack of Internet access and lack of technical support [19,20,22,23]. Another significant finding in 2013 was the association of patient portal use with medication adherence, disease control, self-maintenance of health, and including the patient in the medical decision [16,19,22,23].

Ten studies and one review were analyzed from 2014. Researchers found an increase in communication between patients and provider through SM, as well as an increase in communication between patients and their health system, which resulted in an increase in customer retention through use of the patient portal [27,29]. Patients continued to respond positively about the SM feature of a portal or a portal-like app [24,26,32,33]. Use of the portal increased the number of office visits and phone contacts in one study [28], but in the review published in 2014, de Jong et al reported a decrease in the number of office visits. Last, Zikmund-Fisher et al evaluated portal user access to lab test results [30]. The portal users could not accurately interpret lab results that indicated level of disease management in diabetes patients. They concluded that health literacy and numeracy skills serve as barriers to full utility of the patient portal. If the patient can view the information but cannot interpret the numbers, they would in turn contact their provider for an interpretation, which defeats the goal of the patients being able to interpret their lab results without the provider having to call.

### Bias, Validity, and Reliability

Several studies evaluated did not use randomization nor did they manipulate an independent variable. Studies without randomization of participants run the risk of selection bias, which, in turn, affects the internal validity. The articles reviewed did not provide a discussion section on bias or their efforts to compensate for the same. Non-experimental designs do not manipulate the independent variable (use of the patient portal) on a dependent variable (quality or Meaningful Use). Lack of a strong research design also reduces the internal validity of the study.

The risk of detection bias, or bias in how outcomes are ascertained, should not be low due to a common standard of care for chronic conditions; however, not all studies reviewed empirically measured outcomes. Reports of improved quality were primarily self-reported by users of the patient portal or portal-like apps.

Most studies that we reviewed provided sufficient detail for other researchers to duplicate their research, therefore the reliability of what they measured is strong. In the Methods section, we summarized our search criteria, and in Multimedia Appendix 1 we summarized results and applicability, loosely following the PICOS model identified in the PRISMA checklist. This review took extra care to ensure the consistency of measurement and reproducibility; we summarized the findings of previous studies and reviews, and we related these findings to our research questions pertaining to quality and Meaningful Use. Therefore, the reliability of this review should be acceptable. Unfortunately, our review did not record the specifics from each researcher on article selection. As in the Ammenwerth et al review, articles were reviewed by 2 researchers, and any differences in judgment were resolved by discussion.

## Discussion

### Summary of Evidence

The US Agency for Healthcare Research and Quality (AHRQ) lists several indicators of quality [35]. Most of these indicators surround the management of chronic conditions like diabetes and hypertension, as well as preventative care. The US Health Resources and Services Administration (HRSA) identifies quality improvement initiatives in health care, namely patient satisfaction and including the patient in medical decisions. This review identifies several quality indicators that are generated from both AHRQ and HRSA.

The use of the patient portals in this review illustrates a higher retention rate of patient loyalty [29] and lower appointment no-show rates [9]. Portal users tend to be female, Caucasian, under 65 years old, well educated, and prefer electronic means of communication [8,16,26,29]. Studies documented a high rate of patient satisfaction with the portal, which enables patients to take a more active role in medical decision making [16,17,24]. Sociodemographic disparities exist for portal use, and users need to improve their health literacy in order to better interpret the medical information they are viewing [8,10,16,18,21,30]. Portal use also seems to increase patient-to-provider communication with only a slight increase in workload or office visits [13,15,23,26,28,29]. Results varied on improved outcomes [14,16,24].

Patient portals seem to offer great potential for higher quality care, but it is unknown whether providers who offer the portals will be able to capitalize on the Meaningful Use, stage 2 incentive due to lack of awareness of the patient portal service [24,25,27]. Measure seven of 17 states requires eligible professionals (EP) to “provide patients the ability to view online, download and transmit their health information within four business days of the information being available to the EP” [2]. In this review, there was insufficient data to associate the use of the patient portal with Meaningful Use.

To improve the association of use of the patient portal with Meaningful Use, hospital administrators should focus heavily on the incorporation of training in proper portal use for patients. Portal developers should conduct ease-of-use studies on their products. If the portal is not easy to navigate, it will not be used. Policy makers should consider the extension of Meaningful Use incentives in the area that affects patient portals. The market has been slow to adapt, and as a result, the maturity of the portal is not where it needs to be in order to improve quality of care and more deeply involve the patient in the medical decision.

### Limitations

It is important to stress the broader scope of study design analyzed in this review compared to that of Ammenwerth et al. When our team initially attempted to duplicate the original study, we did not find any RCTs, and we found only one quasi-experimental study. We chose to open the search criteria to observational studies. The results of studies with weaker designs is weaker results to analyze.

A large limitation to this study was the lack of the key term “patient portal” in MeSH. As depicted by Figure 1, we searched for this key term in all three research databases, but this portion of the search in PubMed resulted in an error. We sent a message to the Library of Medicine to call attention to this fact.

As a result of the absence of “patient portal” in MeSH, as well as differences in syntax required by each database, the queries from PubMed and CINAHL were not matched exactly with the same queries from Google Scholar. Boolean search operators were used in PubMed and CINAHL, but Google Scholar does not enable the use of this basic search method.

The limited ability of Google Scholar to filter and save searches could greatly limit the effectiveness of the search itself. Fewer than 2% of the queries on Google Scholar matched the selection criteria for this study, and the search engine’s rudimentary filters forced a manual process of review for inclusion/exclusion criteria. Ammenwerth et al and this study share a common limitation on this issue; we undoubtedly omitted key research in our reviews.

The differences in search strategies for different databases, the absence of filters in Google Scholar, and the manual process of review for the Google Scholar results could easily affect the quality of analysis of this review. A key-journal search could have been used as a form of validation for the Google Scholar results. For instance, from the initial searches, a list of the top three journals that publish material on patient portals could be identified for a targeted search, as we did with the *Journal of Medical Internet Research*. That search would help validate or highlight weaknesses in the search terms used. If results from the key-journal search highlight a significant number of articles that were not picked up by the other queries, then search terms would need to be added to the initial queries.

### Conclusions

This study systematically reviewed literature from January 1, 2011, to August 24, 2014, to assess the outcome of patient portal use and its effect on quality of care and medical outcomes, effectively duplicating the study by Ammenwerth et al. Approximately 89% of papers reviewed were non-experimental, 52% were qualitative, 67% were quantitative, and 22% were mixed-methods. The mixed-method studies reflect those that were both quantitative and qualitative. Only two studies were quasi-experimental, and no studies used the RCT study design. Ammenwerth et al were able to find four RCTs for their study. We did not identify any RCTs.

The Ammenwerth et al review did not identify any improvements in health outcomes, but it analyzed only RCTs. In contrast, this review identified several clinical and administrative improvements that qualify as quality, as defined by the AHRQ and HRSA, but our review did not include any RCTs. Improvements were identified in medication adherence and the management of chronic disease [5,9,13,22], disease awareness [33], improved self-care [19,22], general “clinically relevant benefits” [13], and a decrease in the number of office visits [33]. Use of the patient portal also increased customer retention [29], which is related to continuity of care. The use of the patient portal was also associated with extended office visits to ask questions of the providers, and an increase in preventative medicine [19]. Although each article talked about application features related to Meaningful Use, only one study specifically used the term [24]. Meaningful Use incentives outlined by the HITECH Act provide money to health care organizations for specific adoption and use of HIT. Features of a patient portal would help organizations meet some of the qualifications for the incentives. Specific to this review would be features of the patient portal such as disease management and secure messaging between patient and provider [36].

Future research should focus on use of the patient portal and empirically measured quality indicators such as medical outcomes, medication adherence, and patient satisfaction. Preferably, the study designs should be RCTs, or at a minimum, an experimental design. The Meaningful Use criteria are designed to improve quality and increase patient involvement in the medical decision. Not all EHRs offer a patient portal, but as seen in this review, there are features of portals that are offered as eHealth apps. The patient portal has great potential to meet both intents of Meaningful Use, but there is not sufficient evidence to declare its efficacy.

## Multimedia Appendix 1

Summary of the studies in the review.

## Multimedia Appendix 2

Full spreadsheet with intervention, control, outcome, and population of each study.

## Abbreviations

AHRQ: US Agency for Healthcare Research and Quality
CINAHL: Cumulative Index to Nursing and Allied Health Literature
EHR: electronic health record
EP: eligible professionals
HIT: health information technology
HITECH: Health Information Technology for Economic and Clinical Health
HRSA: US Health Resources and Services Administration
MeSH: Medical Subject Headings
PHR: personal health record
RCT: randomized control trial
SM: secure messaging
