# Supplementary material for: The Effect of Patient Portals on Quality Outcomes and Its Implications to Meaningful Use: A Systematic Review
Source: J Med Internet Res. 2015 Feb 10;17(2):e44. doi: 10.2196/jmir.3171 (PMC4342639; doi:10.2196/jmir.3171)
Supplement: Supplementary file 1 [file jmir_v17i2e44_app1.pdf]

Multimedia Appendix 1. Summary of studies in the review.

Study, design, sample	Results	Applicability
Goel et al [8]. Telephonic survey to collect reasons for not using the patient portal (N=159)	Mean age 51; 48% black, 72% female, 70% ≥college degree; 63% no desire to enroll, 30% negative feelings toward the portal, 8% computer-related barriers; black less likely than white to recognize the self-management capabilities of the portal ($P<.05$).	Need to assist patients to understand the benefits of using the portal, and interventions to reduce racial disparities must address attitudinal barriers and not focus solely on improving access.
Nijland et al [9]. Mixed methods (N=226).	The DiabetesCoach was evaluated over a 2-year period. Despite positive outcomes, usage of the Web app was hindered by low enrollment and nonuse attrition. The main barrier to enrollment was lack of Internet access and poor user-friendliness of the app.	Ease-of-use will enable diffusion of innovation in health care. Three factors most influenced increased use of eHealth technologies: (1) avoiding selective enrollment, (2) making use of participatory design methods, and (3) developing push factors for persistence.

Horvath et al [10]. Retrospective study of patients at Duke's seven medical clinics (N=58,942)	15.7% (9239) were portal enrollees, scheduled 198,199 appointments with an overall no-show rate of 9.9% (19,668). Enrollees were significantly more likely to be female, white, and privately insured compared with nonusers. Large reductions in no-show rates were seen among historically disadvantaged groups: Medicaid holders (OR 2.04 for nonuser/enrollee, 5.6% difference, *P*<.001), uninsured patients (OR 2.60, 12.8% difference, *P*<.001), and black patients (OR 2.13, 8.0% difference, *P*<.001).	Monthly no-show rates across all medical clinics in the system were significantly reduced among patients registered for portal use.
Palen et al [11]. Retrospective study of patients >18 years old who had access to a patient portal.	Significant increase in the per-member rates of office visits (0.7 per member per year, 95% CI, *P*<.001), phone encounters (0.3 per member per year, 95% CI, *P*<.001), per-1000-member rates of after-hours clinic visits (18.7 per 1000 members per year, 95% CI, *P*<.001), ED encounters (11.2 per 1000 members per year, 95% CI, *P*<.001), and hospitalizations (19.9 per 1000 members per year, 95% CI, *P*<.001) for patient portal users versus nonusers.	Patient empowerment could result in greater use of available health care services.

Urowitz et al [12]. Structured interview (N=17 patients and 65 providers).	Four themes were identified from interview responses: facilitators of disease management, barriers to portal use, patient-provider communication and relationship, and recommendations for portal improvements.	Online chronic disease management portals increase patient access to information and engagement in their health care, but improvements in the portal itself may improve usability and reduce attrition.
Delbanco et al [13]. Quasi-experimental design, no randomization (N=105 providers and N=13,564 patient); studies encounters with at least one doctor's note in a primary care setting at 3 large, but independent locations.	11,757 of 13,564 patients (86.78%) with notes available opened at least 1 note, but only 5391 (39.74%) completed a post intervention survey. Surveys reported up to 87% of these patients felt greater control of their care, up to 78% reported greater medication adherence, 36% had privacy concerns, and up to 42% reported sharing notes with others. Volume of electronic messaging between patients and providers increased 5%. Providers reported up to an 8% workload increase to answer patients' questions outside of visits. Of those providers, 21% reported taking additional time to write notes, and 36% reported changing documentation content.	A majority of patients who accessed their doctors' notes and filled out a survey reported positive, clinically relevant benefits and very few concerns about access.

Osborn et al [14]. Mixed methods design of patients with type 2 diabetes (N=75) to study quantitative data to identify differences between portal and non-portal users.	81% (61/75) attended a focus group and/or completed a survey. Portal users tended to be Caucasian/white, have higher income, be privately insured, have more education, and better A1C test results. All results were statistically significant ($P<.05$) except for education, which was mildly significant ($P=.05$).	Patients noted a preference to use the portal to manage prescriptions and medication adherence. More frequent users of the portal were more likely to have better glycemic control.
Lyles et al [15]. Secondary data analysis of survey responses (N=11,518).	52% were registered users, among those 36% used SM. Greater levels of trust were associated with white, Latino, and older patients.	Patient-provider relationships encourage portal engagement.
Wade-Vuturo et al [16]. Mixed-methods, survey tool, non-experimental, qualitative study (N=15)	Participants were 57.1 years old, 65% female, 76% Caucasian/white, 20% African American/black. SM was reported as enhancing patient satisfaction, efficiency and quality face-to-face visits, and access to clinical care outside traditional face-to-face visits. Greater SM use was significantly associated with patients' glycemic control ($P=.29$)	Portal use enabled collaborative decision making about diabetes management.
Nazi et al [17]. Web study (N=688) of Veterans	84% reported positive satisfaction agreeing that the information and services were helpful. 66% agreed that the portal increase quality of care, and 90% agreed that they would recommend the portal to another veteran.	Veterans demonstrated a high rate of motivation to access their own health information and viewed such action as an increase in quality of care.

Ketterer T et al [18]. Cross-sectional, retrospective analysis (N=84,105)	38% (31,360/84,105) enrolled, 26% (21,867) activated the account. Portal enrollment was lower for adolescents, Medicaid recipients, low-income families, Asian or other race, and Hispanic ethnicity, and higher for patients with more office encounters, and presence of autism on the problem list (95% CI).	Sociodemographic disparities exist in portal enrollment/activation in primary care pediatrics. Proximity had a negative effect, number of office encounters and comorbidity had a positive association on portal enrollment.
Zarcadoolas et al [19]. Qualitative study of four focus groups (N=28) with low-education level, English-speaking consumers	Portal users felt a high level of patient engagement / empowerment. These users extended office visits to ask additional questions of their provider. There was also an increase in preventative and overall health maintenance.	Portal users demonstrated enthusiasm about the increased utility and value of their medical encounter when augmented with the portal.
Shimada et al [20]. Cross-sectional, retrospective cohort study of 32 veterans affairs (VA) facilities implementing SM in primary care	Technical assistance (coordinators), computer resources, and leadership support for coordinators were positively associated with increased SM adoption rates. Higher SM use was associated with lower urgent care (UC) rates, early adopters of SM achieved a greater decrease in UC utilization over time than later adopters.	A path of associations linking SM and reductions in UC utilization exists.

Schprechman et al [21]. Cross-sectional study of older adults aged 50-85 years (N=119)	Internet and email use were reported in 78.2% and 71.4% of this sample of patients with heart failure (HF), respectively. Controlling for age and education, higher health literacy predicted email, but not Internet use. Global cognitive function predicted email (P<.001) but not Internet use. Only 45% used the Internet to obtain information on HF.	The majority of HF patients use the Internet and email, but poor health literacy and cognitive impairment may prevent some patients from accessing these resources.
Woods et al [22]. Qualitative (interview) (N=36)	30 patients and 6 family members, all with access to the patient portal for the Veteran's Health Administration, associated several factors with the portal: positive effect on communication with providers, positive effect on self-management of health and chronic conditions, positive effect on the quality of follow-up for test results, and positive effect in patient-involvement in the medical decisions.	Patients and their delegates report positive effects of the patient portal on communication and overall self-care. Patients felt empowered by the capability to view their records through the portal.
Varsi et al [23]. Qualitative study (N=22) of enrollees of a patient portal but did not use the portal.	(1) Enrollees felt they did not need the patient portal and had sufficient access to information elsewhere,(2) they preferred other types of communication such as phone or face-to-face, (3) they were unable to log in from lack of technical support. Patients were satisfied with the opportunity to send messages to health care providers through the portal, even if they did not use the	Portals should be offered to the patients at an appropriate time when the patient needs the service and when they are receptive to information about the service.

	feature.	
Neuner et al [24]. Mixed-methods study of 2 independent data sources to examine patient enrollment in local portal in both primary care and specialty care settings (N=124,379) for 2010-2012	13.2% (16,418/124,379) of patients enrolled in the portal in 2010, and by 2012 enrollment increased to 23.1% (28,731). Median patient access of portal per year was 14 times, with a range of 1-660. Over 93% accessed the system at least twice, 78% accessed the system 4 or more times, and 15.3% accessed the system 50 or more times per year.	Portal users were slightly older and more likely to be female (*P*<.001). Users reported high levels of satisfaction with functioning of the portal; 96% at least satisfied, (66.5% very satisfied, 30.0% satisfied). 87% primary care and 37% other specialist would have met the email-threshold set by Meaningful Use stage 2 requirements.
Turvey et al [25]. Random survey presented to 4% of My Health*e*Vet users (N=18,398) March-May 2012 to assess use and perceived value of the Blue Button feature	33% were current Blue Button users. Beneficiaries valued most (73%) the capability to better understand their health history, 21% of users with a non-VA personal care providers share their health history with that provider, 87% reported that the non-VA provider found the information somewhat or very helpful.	Educational efforts should be used to raise awareness of this valuable feature.

Lam et al [26]. Cross-sectional analysis (N=324)	55.2% under 65, mean age of 48.2 years. 83.9% of respondents preferred electronic means of communication with their provider, and 84.5% found the SM system easy to use, and 83% were satisfied with the messaging system.	Electronic communication platforms must address key issues in consumer education, physician commitment, and adoption of an accessible interface to ensure productive older adult consumer participation.
Hanauer et al [27]. Qualitative content analysis over a 7-year period for patient-initiated amendment requests (N=688)	77.8% requested correction of erroneous data, and 49.7% of all requests were ultimately approved.	The low number of amendment requests may be due to inadequate awareness
Liss et al [28]. Interrupted time series design (N=18,486)	The mean quarterly number of primary care contacts increased by 28% between the pre-PCMH baseline and the post implementation periods, largely due by increased SM, quarterly office visits declined by 8%, 10% increases in SM threads, and phone encounters were associated with increases of 1.25% (95% CI, *P*<.001) and 2.74% increase in office visits (95% CI) in office visits, respectively.	Before and after a medical home redesign, proportional increases in SM and phone encounters were associated with additional primary care office visit for individuals with diabetes.

Garrido et al [29]. Time series retrospective design (N=36,001)	49% of recorded encounters between personal care providers and patients at Kaiser Permanente occur virtually (16% telephonically, 33% SM). Retention rates were 96.9% for patient portal users (95% CI) and 90.5% for nonusers (95% CI, *P*<.001). Portal users 2.6 times more likely to remain members of Kaiser Permanente (95% CI).	Members who used the patient portal were more likely to remain members of Kaiser Permanente than were those who did not use the portal. More than 70% of users prefer access to their health information than to emailing providers.
Zikmund-Fisher et al [30]. Internet survey (N=1817)	Viewing test results with multiple deviations increased the probability of identifying hemoglobin A1c values as outside the reference range (participants with diabetes: OR 1.47, 95% CI 1.12-1.92, *P*=.005; people without diabetes OR 1.50, 95% CI 1.13-2.00, *P*=.005)	Health literacy and numeracy skills serve as barriers to basic understanding of laboratory test result data presented in the EHR.
Hogan et al [31]. Case study, secondary data analysis, interviews, mixed methods (N=955,800)	Technology-assisted access to clinical information represents a significant advance for VA patients. Recommend that the Blue Button (1) consider both processes of care and outcomes, (2) clearly define constructs of focus, (3) examine influencing factors related to the patient population and clinical context, and (4) identify potential unintended consequences.	The Blue Button does not currently enable a relationship of its own use and health-related outcomes. However, its use does meet the intent of Meaningful Use criteria because it involves the patient in medical decisions.

Haun et al [32]. Interviews and mixed methods (N=33)	97% of veterans (31/33) were satisfied with the capabilities of SM. Four domains emerged: (1) perceived benefits of SM, (2) barriers to the use of SM, (3) facilitators for the use of SM, and (4) suggestions for improving SM.	Veterans appreciate the capability of SM with their providers and feel that additional marketing will spread the word about the capability, and proper training could help others overcome barriers to its use.
de Jong et al [33]. Review, (N=15).	Patients' knowledge and self-management of their chronic conditions increased through the use of the portal, and patients greatly appreciated, and used the SM with their providers. Use of the portal decreased office visits in only two studies.	The patient portal enables self-management and increases awareness of chronic conditions. It also increases the involvement of the patient in the medical decision.
Kashiwagi et al [34]. Experimental, prospective study (N=171)	Allowing patients to browse their medical data may reduce the use and improve the effectiveness of glaucoma medication.	The patient portal enables patients to browse their medical data. Based on this study, use of the portal would increase medication adherence and improve outcomes.
